# Hemoglobin Disorders Associated with Neurological Impairment: First Report of ATR-X Syndrome and Recessive Congenital Methemoglobinemia Type II in Tunisia

**DOI:** 10.3390/ijms26104803

**Published:** 2025-05-16

**Authors:** Houyem Ouragini, Emna Bouatrous, Manel Kasdallah, Sonia Nouira, Hamza Dallali, Samia Rekaya, Dorra Chaouachi, Monia Ouederni, Samia Menif

**Affiliations:** 1 Laboratory of Molecular and Cellular Hematology, LR16IPT07, Institut Pasteur de Tunis, University of Tunis El Manar, Tunis 1002, Tunisia; emnabouatrous@gmail.com (E.B.); manelkasdallah7@gmail.com (M.K.); nouira.sonia@gmail.com (S.N.); samyarekaya@gmail.com (S.R.); dorra.chaouachi@pasteur.tn (D.C.); ouederni.moni@gmail.com (M.O.); samia.menif@pasteur.tn (S.M.); 2 Molecular Biology Cell and Biotechnology Department, Higher Institute of Biotechnology of Monastir, University of Monastir, Monastir 5000, Tunisia; 3 Laboratory of Biomedical Genomics and Oncogenetics, LR16IPT05, Institut Pasteur de Tunis, University of Tunis El Manar, Tunis 1002, Tunisia; hamza.dallali92@gmail.com; 4 Pediatric Immuno-Hematology Unit, Bone Marrow Transplantation Center, Tunis 1006, Tunisia

**Keywords:** ATR-X syndrome, recessive congenital methemoglobinemia type II, neurological disease, mutation, WES, diagnosis

## Abstract

Hemoglobin disorders are among the most common inherited diseases worldwide. Their clinical manifestations range from anemia to more severe forms associated with neurological impairments. These complications can result as secondary consequences of the disease’s clinical manifestations or be directly linked to genetic mutations. In this study, we present two families with neurological impairments who were referred to us for complementary hematological and biochemical analyses. Complete blood count, methemoglobin level, and methemoglobin reductase activity were assessed. Molecular analyses were performed using whole-exome sequencing, and the segregation of the identified mutations was confirmed with direct sequencing. Their pathogenicity and conservation were evaluated using various bioinformatics tools. Clinical and hematological findings suggested X-linked alpha-thalassemia/impaired intellectual development syndrome in the first family and recessive congenital methemoglobinemia type II in the second. This was confirmed by the identification of pathogenic mutations *ATRX*: p.Arg2131Gln and *CYB5R3*: p.Ala179Thr, respectively. Although these variants have been previously reported worldwide, they were identified for the first time in our population. Our results contribute to the understanding of the pathogenesis of these rare disorders and provide a basis for diagnosis, treatment, and genetic counseling. The mechanisms by which these mutations contribute to neurological symptoms are discussed.

## 1. Introduction

Hemoglobin (Hb) disorders, or hemoglobinopathies, are genetic conditions affecting the Hb structure or function. They manifest with hematological symptoms, such as anemia and hemolysis, but severe forms are also associated with complex systemic manifestations, including neurological impairments. These complications can significantly impact the clinical course and prognosis of affected individuals, yet their underlying mechanisms remain poorly understood [1,2]. Among these rare forms are the X-linked alpha-thalassemia/impaired intellectual development syndrome, also called ATR-X syndrome, and recessive congenital methemoglobinemia type II (RCM-II). Despite their distinct genetic etiologies, both conditions share common features, such as cognitive impairments and developmental delays [3,4].

ATR-X syndrome (OMIM #301040) is caused by mutations in the *ATRX* gene, located on the X chromosome, and encoding a protein involved in chromatin remodeling. ATR-X syndrome is characterized by intellectual disability, developmental delays, α-thalassemia (a form of hereditary anemia due to the reduced synthesis of α-globin chains), and distinct craniofacial features. Neurological manifestations are a central aspect of the disorder, with affected individuals presenting severe cognitive impairments, hypotonia, and, in some cases, seizures [3,5].

Recessive congenital methemoglobinemia (RCM) (OMIM #250800) is an autosomal disease resulting from mutations in the *CYB5R3* gene, which encodes NADH-cytochrome b5 reductase 3 (NADH-CYB5R3). Its principal role is to reduce formed physiological methemoglobin (MetHb) to functional hemoglobin. Mutations in *CYB5R3* lead to an excess of MetHb, resulting in cyanosis and tissue hypoxia [4]. While RCM type I affects only red blood cells and is limited to hematological symptoms, such as cyanosis, type II (RCM-II) is a more severe form that impacts multiple tissues. Patients with RCM-II present with severe and progressive neurological impairments, including developmental delays, microencephaly, intellectual disability, and movement disorders. In severe cases, seizures and coma may occur [6,7]. 

This report describes these rare conditions with neurological complications in two Tunisian families: one with two brothers affected by ATR-X syndrome, and another with a boy presenting with RCM-II. Molecular diagnosis by whole-exome sequencing revealed the pathogenic mutations: *ATRX*: p.Arg2131Gln and *CYB5R3*: p.Ala179Thr. We discuss the potential mechanism linking Hb disorders to neurological impairments in these diseases, as well as diagnostic challenges posed by the features of these conditions.

## 2. Results

### 2.1. Family 1

#### 2.1.1. Clinical Features

**Figure 1:**
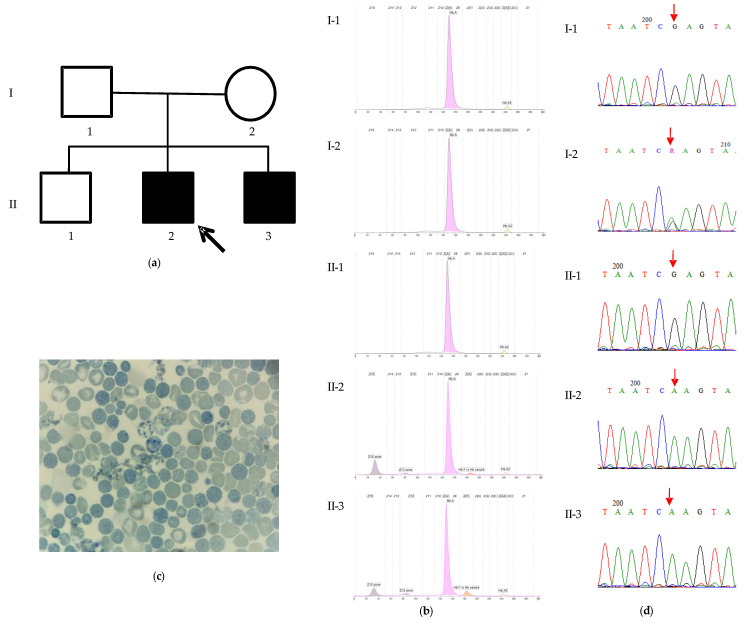
The characteristics of family 1. (**a**) The pedigree of the family and profiles of Hb electrophoresis. The proband (II-2) is indicated by the arrow. (**b**) The proband (II-2): HbA 79.9%, HbA2 1.6%, HbF 1.9%, HbH 15.7%, and Hb Bart’s 1.1%. The proband’s affected younger brother (II-3): HbA 81.4%, HbA2 1.5%, HbF 5.2%, HbH 10%, and Hb Bart’s 1.8%. The father (I-1): HbA 97.5%, HbA2 2.5%. The mother (I-2): HbA 97.6%, HbA2 2.4%. The proband’s healthy older brother (II-1): HbA 97.7%, HbA2 2.3%.; (**c**) A blood smear of the proband revealing HbH inclusion and Heinz bodies; and (**d**) Sanger sequencing chromatograms of the region spanning the *ATRX*: c.6392G>A variant (indicated by a red arrow) from the analyzed family members. The proband (II-2) and his affected brother (II-3) are hemizygous for the variant (A), whereas his unaffected father (I-1) and elder brother (II-1) are hemizygous for the reference sequence (G), and his mother (I-2) is heterozygous (G/A).

The family originated from Sousse, central Tunisia. Both parents are healthy and have three children. The eldest child is a healthy 12-year-old boy, while the two younger male siblings present with severe psychomotor developmental delay and microcytic anemia (Figure 1a).

The proband was admitted at 8 months of age for the evaluation of developmental delay and anemia. On examination, he exhibited microcephaly, hypotonia, facial dysmorphia characteristics of ATR-X syndrome (including slight hypertelorism, a flat nasal bridge, and lower lip evagination), cryptorchidism, and micropenis. His younger brother, who was born 9 months after the proband’s initial evaluation, also presents with dysmorphic facial features, microcephaly, and genital ambiguity. Both brothers experienced recurrent respiratory infections requiring hospitalization.

No history of intellectual disability or consanguinity was reported in the family. Prenatal care was limited, including the absence of nuchal translucency measurements. Neonatal asphyxia was ruled out.

Both brothers exhibit microcytic hypochromic anemia, and presented a similar Hb profile (Table 1, Figure 1b). Both parents and the unaffected elder brother exhibited normal Hb profiles (Figure 1b).

The hematological and clinical presentations of the patient and his younger brother were suggestive of ATR-X syndrome. A blood smear was stained with brilliant cresyl blue, revealing the presence of HbH inclusion and Heinz bodies in red blood cells (Figure 1c).

#### 2.1.2. Mutation Analysis

The mutational analysis by whole-exome sequencing (WES) identified a previously described pathogenic variant, p.Arg2131Gln (NM_000489, c.6392G>A), located in exon 29 of the *ATRX* gene. The subsequent Sanger sequencing confirmed this variant in a hemizygous state in the affected brother. The mother was found to be a heterozygous carrier, confirming maternal transmission, while the father and unaffected sibling were negative for the variant (Figure 1d).

The pathogenicity of the variant was confirmed by SIFT, Polyphen-2, PANTHER, PhD-SNP, SNPs&GO, and Mutpred2. The stability of the mutant ATRX protein was predicted to be decreased by I-Mutant and MUpro (Table 2).

**Figure 2:**
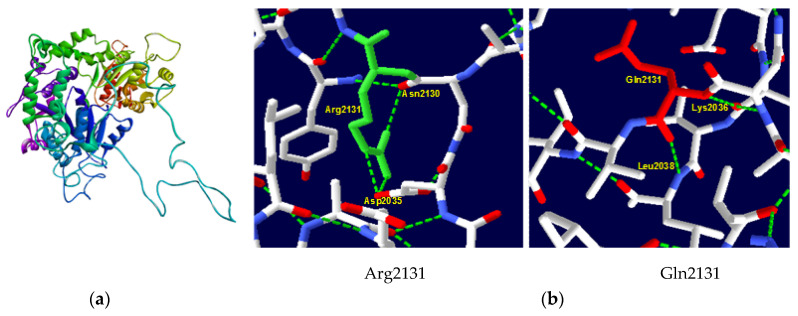
ATRX protein 3D structure. (**a**) Native structure of ATRX protein generated by SWISS–MODEL server; (**b**) structure of wild-type Arg2131 and mutant type Gln2131. Substitution in codon 2131 eliminated hydrogen links with Asp2035 and Asn2130 and gained hydrogen links with Lys2036 and Leu2038.

Owing to the substantial length of the ATRX protein (2492 amino acids), comprehensive molecular modeling was not feasible. Consequently, our template selection was restricted to those encompassing the region harboring the Arg2131 mutation. A 9BZ0 template (Electronic microscopic structure) was used to generate our native and mutant protein structures in the SWISS–MODEL server. It had a 32.21% sequence identity and 21% coverage. The native structure of the protein is shown in Figure 2a. A significant modification in the hydrogen bonding interactions of amino acids was observed between the native and mutant protein (Figure 2b). In the wild-type protein, the amino acid Arg2131 forms crucial hydrogen bonds with Asp2035 and Asn2130. The substitution with Gln results in a new hydrogen bond with Lys2036 and Leu2038 which can potentially destabilize the protein’s secondary structure.

### 2.2. Family 2

#### 2.2.1. Clinical Features

The proband, a 10-year-old boy, was born full term with a low birth weight of 2050 g. He presented at birth with congenital cyanosis and dyspnea. Cardiorespiratory etiology was ruled out. A significant psychomotor delay was observed, including delayed walking at 24 months, intellectual disability, and speech delay. Behavioral disturbances manifested as school difficulties and concentration problems, requiring pediatric psychiatry follow-up. A history of generalized seizures was reported. The brain MRI was unremarkable. Despite episodes of variable cyanosis associated with elevated MetHb levels, the patient did not require intensive care and responded well to treatment with methylene blue, riboflavin, and vitamin C.

**Figure 3:**
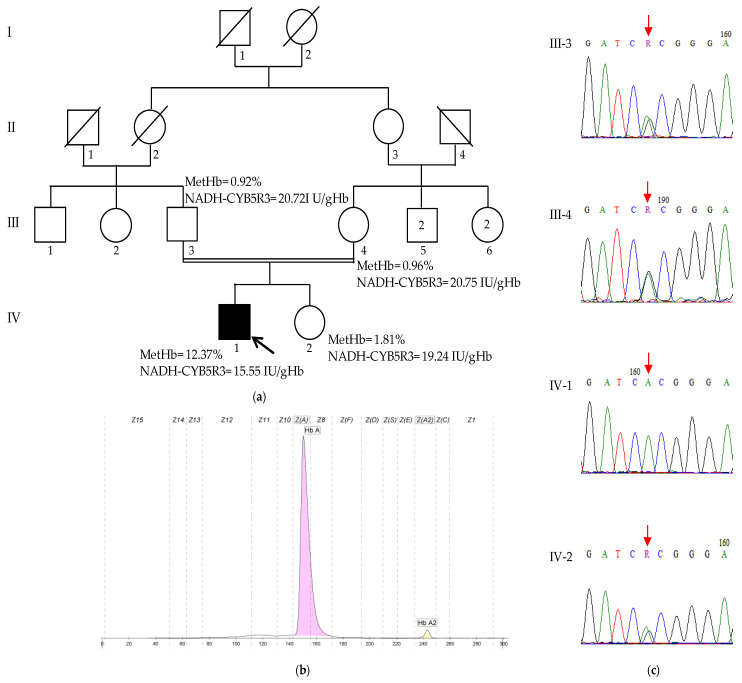
The characteristics of family 2. (**a**) The pedigree of family 2, MetHb levels, and NADH-CYB5R3 activities. The proband (IV-1) is indicated by the arrow; (**b**) the Hb profile of the proband (IV-1): HbA 97.8%, HbA2 2.2%; and (**c**) Sanger sequencing chromatograms of the region spanning the c.535G>A variant (indicated by a red arrow) from the analyzed family members. The proband (IV-1) is homozygous for the variant (A/A), whereas his unaffected parents (III-3 and III-4) and his unaffected sister (IV-2) are heterozygous (G/A).

A history of a second-degree consanguinity was reported, but no other family members exhibited similar conditions (Figure 3a).

The methemoglobin levels were significantly elevated at 12.37% of total hemoglobin (normal value < 2%); the Hb electrophoresis was normal, with HbA and HbA2 levels within reference ranges (HbA: 97.8%, HbA2: 2.2%) (Figure 3a,b). The NADH-CYB5R3 activity was decreased at 15.55 UI/gHb (normal reference value > 19.19 UI/gHb). Neither the patient’s parents nor his healthy sister exhibited elevated methemoglobin levels or decreased NADH-CYB5R3 activity (Figure 3a). To further elucidate the cause of the patient’s condition, genetic testing was performed.

#### 2.2.2. Mutation Analysis

WES revealed a homozygous missense variant in the exon 6 of the *CYB5R3* gene, c.535G>A (NM_000398.7), resulting in a p.Ala179Thr (NP_00387) amino acid change, which has been previously reported. Sanger sequencing confirmed the presence of this variant in a heterozygous state in both parents and the healthy sister, supporting an autosomal recessive inheritance pattern (Figure 3c). 

All the functional tools used predicted that the variant was pathogenic, and the stability of the mutant protein was predicted to be decreased by both the tools I-Mutant and MUpro (Table 2).

**Figure 4:**
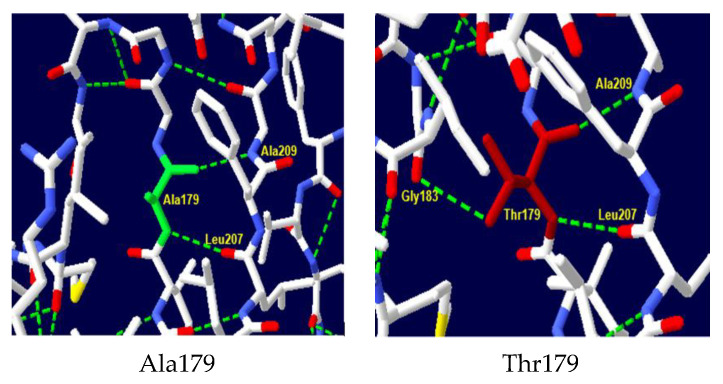
NADH-CYB5R3 protein 3D structure. Structure of wild-type Ala179 and mutant-type Thr179. Substitution in codon 179 added extra hydrogen link with Gly183.

A comparative structural analysis of the wild-type and mutant protein using Swiss-PDB Viewer software v4.10 revealed significant alterations in the hydrogen bonding network within the protein’s active site (Figure 4). In the wild-type protein, the amino acid Ala179 forms critical hydrogen bonds with Leu207 and Ala209, stabilizing the protein’s tertiary structure and contributing to the formation of the active site. The substitution of Ala179 with Thr in the mutant protein disrupts these bonds and leads to the formation of a new hydrogen bond with Gly183, which can have significant functional consequences, including changes in protein stability, folding, and ligand binding affinity.

## 3. Discussion

Hb disorders are among the most common inherited diseases worldwide. The clinical manifestations range from anemia, jaundice, and splenomegaly to more severe complications, such as growth delays, skeletal abnormalities, and organ damage, reflecting the high degree of phenotypic variability in these disorders [8,9]. Hemoglobinopathies with neurological involvement are rare but significant. For instance, SCD patients frequently experience cerebrovascular complications, such as ischemic stroke, which can lead to severe sequelae, including motor deficits and cognitive disorders [10]. In beta-thalassemia major patients, iron overload, a common complication of the disease, can further exacerbate neurological damage through oxidative stress and the generation of free radicals [11,12]. Other manifestations include seizures, anxiety, and depression [10,13,14]. All these neurological impairments are secondary complications of the disease’s clinical symptoms. In contrast, in ATR-X syndrome and RCM-II neurological manifestations are more directly linked to genetic mutations. The exact mechanisms by which these mutations lead to these symptoms remain unclear and are thought to be a complex interplay of genetic, epigenetic, and environmental factors.

In this study, we report the first documented cases of ATR-X syndrome and the third case of RCM-II in the Tunisian population. These patients were referred to us for hemoglobin electrophoresis and methemoglobin quantification. Given their analyses results and their clinical presentations, we carried out molecular analyses that identified pathogenic mutations in each case: the p.Arg2131Gln mutation in the two ATR-X syndrome-affected brothers in family 1 and the p.Ala179Thr for the RCM-II patient in family 2. Although these mutations have been previously reported worldwide, they were identified for the first time in our population. 

The ATRX protein is a member of the SWI/SNF2 family of chromatin remodeling factors. It plays a central role in the maintenance of genome stability and function, such as the regulation of the chromatin state, gene expression, and DNA damage repair. Mutations in the *ATRX* gene can result in ATR-X syndrome or in many types of cancer, particularly in glioma [15]. To date, more than 190 *ATRX* mutations in 200 patients with ATR-X syndrome have been reported worldwide [5]. The Arg2131Gln substitution has been previously reported in a patient with developmental delay, hypotonia, and dysmorphic features, like our patients [16]. However, some clinical differences are observed: our patients present microcephaly, genital anomalies, as well as microcytic hypochromic anemia, while the patient reported by Lee et al. has cardiac malformation and cerebral palsy. Although ATR-X syndrome is characterized by some clinical features, like facial dysmorphias, hematological abnormalities, and intellectual disability, only the latter has been reported in all the affected individuals [5,17]. This demonstrates the clinical heterogeneity of the disease, even in the same family, and the complexity of its pathophysiological mechanism. Although mutations occurring in the ADD domain of the protein have been suggested to be associated with more severe psychomotor phenotypes (such as intellectual disability, hypotonia, or delayed motor development) and mutations in the C-terminal domain may lead to genitourinary impairments, genotype/phenotype correlation could not clearly established, due to the low number of cases reported worldwide [18,19]. All these data suggest that the effect of the abnormal ATRX protein may be influenced by other genetic and/or epigenetic factors [20].

The Arg2131Gln is located at the C-terminal ATPase/helicase highly conserved domain. This domain is essential to the recruitment of ATRX to heterochromatin in neuronal cells [21]. The abnormal protein probably suppresses the expression of several genes in these cells, leading to malformations and intellectual disability [22]. Among these genes, *HBA1*/*HBA2* genes, encoding for alpha globin chains, are also repressed. Indeed, this cluster is a GC-rich locus, and it is known that ATRX regulates gene expression in these particular regions. In the absence of ATRX, there is an increased level of the histone variant macroH2A at the alpha–globin locus, which generally represses the transcription [23]. This could explain the alpha-thalassemia, observed in the ATR-X syndrome. On the other hand, it has been demonstrated that the lack of the normal ATRX protein has negative consequences on the forebrain formation and neural progenitor cells, making it essential for normal development [5]. In addition, as the protein is implicated in the regulation of DNA damage response and the chromosomal instability prevention [15], the increased genomic instability may contribute to the progressive nature of the neurological phenotype. 

Recessive congenital methemoglobinemia is caused by mutations in the *CYB5R3* gene, which encodes NADH-CYB5R3. A total of 82 pathogenic variants have been described so far. Among them, 34 mutations have been reported to be associated with RCM-II [24]. These mutations lead to altered splicing, the disruption of the enzymatic active site, or the premature truncation of the protein [25]. Our patient presents a p.Ala179Thr mutation located in the highly conserved C-terminal/NADH-binding domain. This is the third Tunisian case of RCM-II and the first one with a substitution; the two others harbor splice site mutations [7,26]. The p.Ala179Thr mutation was described for the first time in a North African family, and it is considered the most frequent mutation in the Indian population. All the reported patients were affected by the type I of RCM, unlike our case [27,28]. In general, *CYB5R3* mutations are associated with only one form of the disease. Only three variants have been reported with both type I and type II of RCM: p.Val253Met and Gly76Ser and Leu188del [24,29]. These data suggest the presence of other modifier loci which contribute to the presence of a particular phenotype.

In RCM-II, the two NADH-CYB5R3 variants are abnormal: (1) the soluble erythrocyte isoform, involved in the electron-transport system for reducing methemoglobin to functional hemoglobin able to transport oxygen and (2) the membrane-bound isoform, anchored to the mitochondrial outer membrane, endoplasmic reticulum, and plasma membrane of somatic cells, involved in several metabolic pathways, including fatty acids desaturation and elongation, cholesterol biosynthesis, and cytochrome P450-dependent drug metabolism [30,31]. All these processes are important for normal brain development. For a long time, it was hypothesized that RCM-II caused neuronal demyelination [32]. However, this alone is insufficient to explain the etiology of this disease. Recently, the NADH-CYB5R3 protein has been reported as playing a role in multiple other functions, such as in the mitochondrial homeostasis in neurons and cardiomyocytes, NAD+/NADH regulation, synaptic plasticity, and the vascular vasodilatation response by soluble guanylate cyclase and protein kinase G-dependent signaling. The cumulative effect of these altered mechanisms could contribute to the neurologic symptoms of RCM-II [33].

Hereditary neurological diseases include several diseases with significant clinical variability, which are associated with high genetic variability. Because of this heterogeneity, diagnosis is challenging. A correct identification of the pathology is crucial for patients and their families, providing appropriate treatment and information on the prognosis of the disease. Receiving a definitive diagnosis has a significant psychological impact, as it marks the end of the “diagnostic Odyssey” [34], and this process is now faster thanks to new technologies, such as next-generation sequencing (NGS). In this case, a detailed clinical and biological examination of the patient is necessary to support the analysis of the data generated by NGS. Indeed, some characteristics are exclusive to specific diseases or are only associated with a certain number of specific genes. In our study, the patients presented not only psychomotor developmental delay but also alpha-thalassemia for one and methemoglobinemia for the other. This allowed us to focus on ATR-X syndrome and recessive methemoglobinemia, respectively. These findings highlight the need for increased awareness among healthcare providers regarding the phenotype heterogeneity of rare disorders. 

The lack of detailed clinical information on the seizure course of patient 2 (including age of onset, duration, frequency, and treatment) and the absence of patients’ photos (particularly for patient 1’s facial dysmorphism) represent a limitation of our study. This restricts a comprehensive understanding of the variant impacts. Moreover, a detailed description of patients affected by these rare conditions would facilitate the establishment of a genotype–phenotype correlation by enabling comparisons with cases reported in studies worldwide.

## 4. Materials and Methods

The study was conducted in accordance with the Declaration of Helsinki and approved by the Pasteur Institute’s Biomedical Ethics Committee, Tunis, Tunisia (no.: 2023/09/I).

### 4.1. Clinical Data and Sample Collection

Written informed consent was obtained from the patients’ parents for blood samples, genetic analysis, and publication of clinical and molecular data. Unfortunately, we did not obtain the consent for the patients’ photos.

Clinical details were obtained from the patients’ medical files. Whole blood samples were obtained from each participant into a 5 mL EDTA tube.

Hematological parameters were obtained using an automated blood cell counter (Coulter Counter ABX Micro-60-OTR; ABX Diagnostics, Montpellier, France) to perform a cell blood count. The MetHb level and the NADH-CYB5R3 activity were measured by spectrophotometery at 633 nm and 320 nm, respectively, as previously described [35]. The Hb profiles were determined using capillary electrophoresis (Capillarys 2, SEBIA, Lisses, France). DNA was extracted by the salting out method. Concentrations and purity were measured using the Agilent TapeStation gDNA Screen Tape (Santa Clara, CA, USA).

### 4.2. Molecular Analysis

WES was performed at Macrogen (Seoul, Republic of Korea). Briefly, exome capture was carried out using Twist Bioscience Human Core Exome, and high-throughput sequencing was then performed using the Illumina NovaseqX platform (San Diego, CA, USA) to generate 150 bp paired-end reads.

The identified variants in *CYB5R3* and *ATRX* genes were validated by Sanger sequencing performed on automatic sequencer ABI PRISM 310 Genetic Analyzer (Applied Biosystems, Foster City, CA, USA). Briefly, PCR was carried out in a 25 µL reaction containing 50 ng of DNA, 10 pmol of each primer, 1× buffer, 0.2 mM dNTP, 2 mM of MgCl_2_, and 0.04 U of Taq polymerase (Invitrogen, Carlsbad, CA, USA). After an initial denaturation at 95 °C for 5 min, 35 cycles were performed (95 °C for 30 s, 64 °C for 1 min, and 72 °C for 1 min), followed by a final extension for 7 min at 72 °C. The F-5′ TGTATTATCATCCTGACTCC 3′, R-5′ TTTCCAACTTTGTTTCCCTC 3′ primer set was used for amplifying the exon 29 of *ATRX* gene; and the F-5′ CCTCTACCTCGGACCTCACA 3′, R-5′ GTCATCCCCAGAATCTCAGC 3′ primer set was used for amplifying the exon 6 of *CYB5R3* gene.

### 4.3. Bioinformatics Analyses

Genetic analysis of the paired-end sequencing data was performed using an in-house bioinformatics pipeline. Sequencing reads were aligned to the GRCh38/hg38 reference genome using the BWA-MEM tool v1.1.1, after checking the quality of the raw data with FastQC program v0.12.0. The resulting BAM files were processed using GATK best practices. Duplicates were marked using GATK MarkDuplicates v4.4.0.0, following by an application of base score quality recalibration using the GATK BQSR algorithm v4.4.0.0. Then, variants were named using GATK HaplotypeCaller v4.4.0.0 to produce GVCF files, which were subsequently annotated using the VarAFT v2.16 tool to prioritize variants based on their functional impact and association with knowing diseases.

Variants which had a read depth below 10 and with a minor allele frequency greater than 1% in 1000 Genomes Project datasets or in genome Aggregation database (https://gnomad.broadinstitute.org/) (accessed on 11 December 2023) were discarded. Only nonsense, missense, indels, and splicing-site variants were retained. The impact of the variants on the protein function was analyzed using several in silico prediction tools, such as SIFT https://sift.bii.a-star.edu.sg/ (accessed on 12 December 2023), Polyphen-2 http://genetics.bwh.harvard.edu/pph2/ (accessed on 12 December 2023), PANTHER http://www.pantherdb.org/tools/ (accessed on 12 December 2023), PhD-SNP http://snps.biofold.org/phd-snp/phd-snp.html (accessed on 12 December 2023), SNPs&GO https://snps.biofold.org/snps-and-go/snps-and-go.html (accessed on 12 December 2023), and Mutpred2 http://mutpred.mutdb.org/#qform (accessed on 12 December 2023). The effect of splice-site variants was evaluated by Human Splicing Finder https://bio.tools/human_splicing_finder (accessed on 13 December 2023). The effect of the missenses on protein stability was analyzed through I-Mutant http://folding.biofold.org/i-mutant/i-mutant2.0.html (accessed on 13 December 2023) and MUpro Server http://mupro.proteomics.ics.uci.edu/ (accessed on 13 December 2023).

Wild-type and mutant protein structures were visualized by the PyMOL software v.2.5.2 (https://pymol.org/2/), using the template PDB ID: 1UMK for NADH-CYB5R3 protein. For ATRX protein, as no crystal structure is available, homology modeling using SwissModel was conducted to predict its 3D structure. This server identifies closely related sequences as templates for structure prediction by aligning them with the input query sequence.

Hydrogen bond changes for native and mutant proteins were assessed by using the Swiss-PDB Viewer software v4.10.

## 5. Conclusions

This study not only expands the clinical and genetic landscape of ATR-X syndrome and RCM-II but also provides new insights into the broader implications of Hb disorders on neurological health. Even though there is no treatment for the neurological impairments associated with these diseases, the identification of the pathogenic mutations is crucial for diagnosis, understanding the genetic basis of these rare conditions, and enabling appropriate genetic counseling and management for affected patients and their families.

## Figures and Tables

**Table 1:** The hematological characteristics of the proband and his affected brother.

	The Proband (II-2)	The Affected Brother (II-3)
Hb (g/dL)	9.3	7.9
MCV (fL)	58	56
MCH (pg)	13.4	15.3
HbA (%)	79.9	81.4
HbA2 (%)	1.6	1.5
HbF (%)	1.9	5.2
HbH (%)	15.7	10
Hb Bart’s	1.1	1.8

Hb: hemoglobin; MCV: mean corpuscular volume; and MCH: mean corpuscular hemoglobin.

**Table 2:** Pathogenicity prediction scores of the identified variants.

Mutations (Gene)	Functional Impact Analysis						Stability Analysis	
	SIFT	PolyPhen-2	PANTHER	PhD-SNP	SNPs&GO	Mutpred2	I-Mutant	Mupro
p.Arg2131Gln (*ATRX*)	deleterious	probably damaging	probably damaging	Disease	Disease	Pathogenic	Decreased	Decreased
p.Ala179Thr (*CYB5R3*)	deleterious	probably damaging	probably damaging	Disease	Disease	Pathogenic	Decreased	Decreased

## Data Availability

The original contributions presented in this study are included in the article. Further inquiries can be directed to the corresponding author.

## Abbreviations

The following abbreviations are used in this manuscript:

Hb	Hemoglobin
RCM	Recessive congenital methemoglobinemia
RCM-II	Recessive congenital methemoglobinemia type II
NADH-CYB5R3	NADH-cytochrome b5 reductase 3
MCH	Mean corpuscular hemoglobin
MCV	Mean corpuscular volume
MetHb	Methemoglobin
WES	Whole-exome sequencing
SCD	Sickle cell disease
NGS	Next-generation sequencing
